# Copper at the Front Line of the Host-Pathogen Battle

**DOI:** 10.1371/journal.ppat.1002887

**Published:** 2012-09-20

**Authors:** Richard A. Festa, Dennis J. Thiele

**Affiliations:** Department of Pharmacology and Cancer Biology, Duke University Medical Center, Durham, North Carolina, United States of America; Duke University Medical Center, United States of America

## Introduction

Copper (Cu) is a transition metal used by life from bacteria to eukaryotes in many cellular processes as a biochemical cofactor and a signaling molecule. However, while Cu plays critical cellular roles, it can be toxic when allowed to accumulate to levels well beyond cellular needs. This razor's edge between the essentiality and toxicity of Cu is emerging as a critical host defense mechanism at the heart of the host-pathogen axis. Accumulating evidence suggests that the innate immune response commandeers the toxic properties of Cu to attack invading infectious organisms, while pathogenic bacteria and fungi have implemented robust mechanisms for Cu resistance. The fact that Cu resistance mechanisms are frequently found among pathogens, and required for virulence, suggests that this is an important aspect of survival in the host. Here, we suggest answers to four fundamental questions about our current understanding of the role of Cu in microbial pathogenesis.

## Why Is Cu Both Useful and Toxic in Biological Systems?

Cu is a useful cofactor for enzymes due to its ability to redox cycle between reduced (Cu^+^) and oxidized (Cu^2+^) forms. Cu^+^ and Cu^2+^ have affinity for cysteine and methionine, or aspartic acid, glutamic acid, and histidine, respectively, allowing for a plethora of distinct biochemical interactions [1]. One essential and conserved Cu-dependent process in many aerobic forms of life, especially in Eukaryotes, is oxygen reduction by cytochrome *c* oxidase, leading to the production of ATP. While Cu functions in this and other essential processes, free intracellular Cu can lead to oxidative stress, caused by Fenton chemistry, whereby cycles of Cu oxidation and reduction produce reactive oxygen species such as the hydroxyl radical. Furthermore, Cu can disrupt iron-sulfur clusters and displace other metals from their cognate enzymes, leading to inactivation [2]. Evidence suggests that free Cu in the cytoplasm is maintained at less than one atom per cell, highlighting the ability of cells to tightly regulate Cu within a given homeostatic range [3].

## How Do Hosts Use Cu to Fight Off Invading Pathogens?

**Figure 1:**
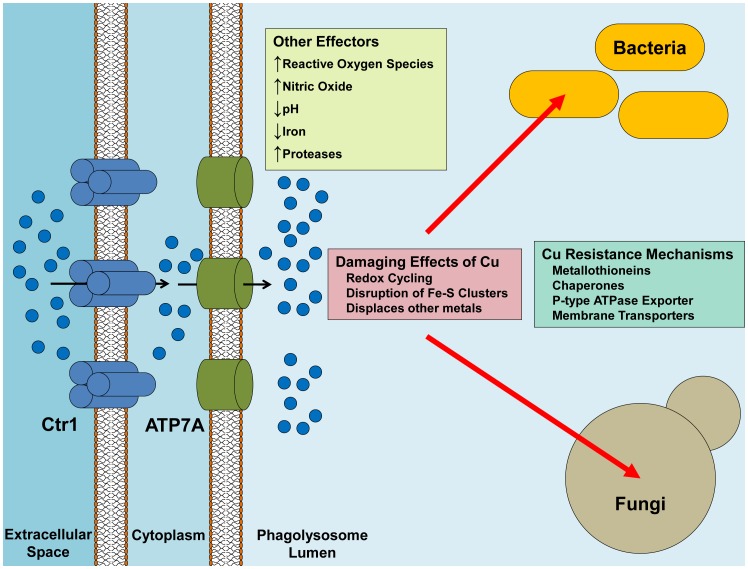
Delivery of Cu from the macrophage to the pathogen. Ctr1 is induced upon activation of the macrophage, possibly leading to increased Cu uptake by the macrophage. Similarly, ATP7A expression is increased and the protein is re-localized to the phagosomal membrane, with the net effect being an increased concentration of Cu in the phagolysosomal lumen. Cu can synergistically react with other effectors such as the production of ROS and NO to promote more oxidative damage. Pathogenic bacteria handle Cu-toxicity mostly through sensing and export mechanisms, while some bacteria have buffering capabilities by binding Cu in chaperones and metallothioneins. Pathogenic fungi, on the other hand, have a higher requirement for Cu usage, but still maintain robust mechanisms for Cu detoxification.

It has long been known that Cu deficiency in mammals compromises immune cell function and sensitizes hosts to microbial infection [4]. While the precise mechanisms for Cu-facilitating immune responses are not well understood, some inroads have recently been made through studies in macrophages. These phagocytic innate immune cells are often the first line of defense against pathogens. Upon activation and phagocytosis of a pathogen, the phagolysosome transitions to a highly anti-microbial milieu with an acidified pH, the generation of reactive nitric oxide and oxygen species, elevation of luminal protease levels, and the mobilization of iron, a well-established player in microbial virulence, out of the lumen and into the cytoplasm. Previous evidence, gained through the use of X-ray microprobe analysis, suggests that in response to infection of peritoneal macrophages with infectious *Mycobacterium* species, there is a time-dependent accumulation of Cu within the phagosome [5]. Recent work in the Petris laboratory has shown, in both macrophage-like cell lines and in primary macrophages, that levels of the Ctr1 Cu^+^ importer are elevated in IFN-γ and LPS-activated macrophages [6]. Furthermore, the steady state protein levels of the ATP7A Cu pump, which delivers Cu to the secretory compartment or exports Cu from most cell types, is elevated and ATP7A is partially localized to the phagolysosome, suggesting that the phagolysosomal accumulation of Cu is due to the recruitment of these two Cu transporters (Figure 1). Consistent with these observations, *E. coli* mutants defective in Cu resistance are hypersensitized to macrophage killing and, conversely, chelation of macrophage Cu resulted in increased survival by *Salmonella typhimurium* [6], [7]. Taken together with our knowledge of the toxic properties of excess Cu, these early observations suggest that Cu may be a significant part of the anti-microbial arsenal within the lumen of the phagosome. Furthermore, Cu can chemically react with ROS and NO, potentially amplifying their toxic effects. Identifying the role of Cu in other immune cell types, such as neutrophils, microglia, dendritic cells, and natural killer cells, may provide further insight into the diverse ways the immune system might use Cu as an anti-microbial weapon.

Although activated macrophages express elevated levels of Ctr1 and ATP7A, presumably to target Cu into the phagosome, the source of this Cu is unknown. One possibility is that Cu is provided through the plasma and there is evidence for increased plasma Cu during inflammation as well as at focal points of infection [8], [9]. In the plasma, ceruloplasmin, a Cu containing ferroxidase, is the most abundant Cu-bound protein in the blood and may account for some of this increase. However, while ceruloplasmin is an acute phase protein where plasma levels are increased in the serum during infection and inflammation, a direct link between ceruloplasmin and the provision of Cu to innate immune cells during infection has not been made. Alternatively, immune cells may harbor internal Cu stores that are mobilized during infection. Modulating Cu movement through the periphery and enhancing its availability to macrophages may be one point of intervention in controlling microbial pathogens.

## How Do Bacterial Cells Use and Detoxify Cu?

While most bacteria use Cu, trace Cu is sufficient for survival as there are few Cu-dependent enzymes and they are most often localized within the cell periphery, presumably to prevent toxicity in the cytoplasm. The most widespread Cu-dependent enzyme in bacteria is cytochrome *c* oxidase (Cox), located in the cell membrane [10]. Cu,Zn Superoxide dismutase (SOD) is another cuproenzyme found in pathogenic bacteria that is crucial to disproportionate superoxide anion into oxygen and hydrogen peroxide. In contrast to the manganese- or iron-containing SOD, commonly found in the cytoplasm, Cu,Zn SOD is secreted or associated with the periplasm, obviating the need to load the enzyme with Cu in the cytoplasm [11]. Cu,Zn SOD provides one mechanism to combat oxidative stress and is important for successful infection by a number of pathogens [12]. While Cu bound to methanobactin, a Cu-binding/scavenging small peptide, has been demonstrated to serve as a substrate for Cu import in methanotrophs, to date there are no identified Cu importers or Cu scavenging proteins identified in pathogenic bacteria [13]. Consequently, bacterial cells may prevent Cu toxicity in part by keeping Cu compartmentalized in the cell periphery.

To combat elevated levels of Cu, bacterial pathogens implement sophisticated mechanisms for Cu sensing and detoxification (reviewed in [14], [15]). Cu resistance mechanisms are commonly encoded in an operon adhering to the following basic framework: a Cu metalloregulatory transcription factor (commonly a repressor), a Cu-binding protein (chaperone) that delivers Cu to the repressor, and a P-type ATPase (an exporter homologous to eukaryotic ATP7A). This operon is de-repressed in the presence of Cu, leading to expression of one or more Cu efflux pumps. Gram-positive, Gram-negative, and acid-fast pathogens have modified this basic system to respond to their unique niche.

The (Copper resistance) *cop* operon of the Gram-positive pathogen *S. pneumoniae* is induced in the lungs and nasopharynx of mice during infection and *cop* mutants are correspondingly less virulent compared to wild type strains [16]. Interestingly, some *Staphylococcus aureus* strains, while encoding the canonical Cu response operon, also contain a plasmid encoding further Cu resistance genes under the control of the genomic CsoR repressor, suggesting that Cu hyper-resistance may be able to be transferred between strains [17].

Disruption of the Cu export ATPases of Gram-negative bacteria such as *E. coli* and *S. enterica* leads to enhanced killing in macrophages [6], [18]. The periplasm of Gram-negative bacteria provides a physical buffer between the cytoplasm and the environment and contains multicopper oxidases and other Cu chaperones that protect against Cu stress. Indeed, CueO, the multicopper oxidase of *S. enterica* serovar Typhimurium, is crucial for systemic virulence in the murine infection model [19].

The acid-fast bacterium *Mycobacterium tuberculosis* (Mtb) is a pathogen that is well-adapted to respond to anti-microbial Cu in the host, as shown by its success as a pathogen. The Mtb genome encodes two Cu-responsive regulons, the canonical Cu responsive operon regulated by CsoR [20] as well as a second Cu-responsive regulon controlled by RicR [21]. The CsoR operon is expressed in mice, implicating an important role during infection, and mutation of the CsoR operon member encoding the Cu transporting ATPase, CtpV, leads to decreased lung damage in guinea pigs and a compromised immune response in mice [22], [23]. Moreover, loss of an outer membrane channel protein, MctB, which is not regulated by CsoR or RicR, leads to increased cellular Cu load and decreased virulence in guinea pig infections [24]. In comparison, MymT, a metallothionein, is protective against Cu stress in vitro, but mutation of *mymT* does not result in a detectable decrease in virulence in the mouse infection model. Metallothioneins are small cysteine-rich metal binding proteins that play a critical role in metal detoxification that are more commonly found in Eukaryotes than bacteria [25]. It is unclear why the *mymT* mutant does not have any discernible phenotype in the mouse model, but perhaps using the guinea pig model, where phenotypes were observed for *ctpV* and *mctB* mutants, may elucidate a role.

The universality of Cu resistance across a spectrum of bacterial pathogens, coupled to the notions that no known *bona fide* Cu importers have been identified and there are very few Cu-dependent enzymes in pathogenic bacteria, suggest that pathogens primarily face Cu toxicity, rather than scarcity, during infection. Attacking bacterial Cu resistance mechanisms as an “Achilles heel,” in conjunction with conventional antibiotics, may prove to be a valuable avenue for the development of anti-bacterial therapies.

## How Do Fungi Use and Resist Cu?

As eukaryotic organisms, fungal pathogens share much of the dedicated Cu homeostasis machinery used by same mammalian hosts that they infect. Indeed, our knowledge of Cu homeostasis in mammals has been largely elucidated in studies using the baker's yeast *S. cerevisiae*. Fungi import Cu and safely shuttle it to cytosolic proteins and the mitochondria, the secretory compartment, and cytosol through the use of dedicated Cu importers, chaperones, and pumps [26]. *Cryptococcus neoformans* is an important fungal pathogen, and there are clear indications that Cu homeostasis is important for virulence, highlighting the need to understand Cu metabolism in this organism. Two Cu-dependent proteins, Cu, Zn SOD and laccase, which function in oxidative stress protection and melanin production, respectively, have been demonstrated to play roles in fungal virulence. Moreover, the acquisition of Fe, a known virulence factor, requires a Cu-dependent ferroxidase [27]. Accordingly, previous work has shown that Ctr4, a Cu importer, is expressed when the fungus disseminates and infects the brain [28]. The *C. neoformans* Cu metalloregulatory transcription factor Cuf1, required for expression of the Ctr4 Cu importer under Cu deficiency conditions, was shown to be a virulence factor [28]. However, recent studies demonstrating that Cuf1 also activates expression of two metallothionein genes, *MT1* and *MT2*, under high Cu conditions indicate that further investigations are merited to evaluate whether Cu uptake, Cu detoxification, or both contribute to virulence [29]. Additionally, it will be interesting to identify potential distinct roles for the Cu homeostasis machinery as *Cryptococcus* disseminates from the lung to the central nervous system, which may exhibit different Cu availability, perhaps requiring both acquisition and detoxification for maximal pathogenesis. Other pathogenic fungi such as *Candida*, *Histoplasma*, and *Aspergillus* species encode similar Cu homeostasis machinery as is found in *C. neoformans*, and future studies investigating the role of Cu during infection with these pathogens will be required for a more complete picture of the role of Cu in fungal pathogenesis.

## Thoughts Going Forward

Mounting evidence suggests that the anti-microbial properties of Cu are used by host immune cells as one tool in their arsenal to defend against microbial pathogens. It will be interesting to determine if cell types other than macrophages that interact with pathogenic organisms at the front line, such as enterocytes or neutrophils, similarly take advantage of the anti-microbial properties of Cu. In turn, pathogenic microorganisms implement tightly controlled Cu homeostatic mechanisms to utilize Cu yet resist Cu toxicity. Understanding how the host controls this metal, as well as how pathogens sense and cope with Cu deficiency or toxicity at distinct times after infection and in specific host tissues, may lead to new ideas for the development of novel therapeutic strategies.
